# Acinic Cell Carcinoma in the 21st Century: A Population-Based Study from the SEER Database and Review of Recent Molecular Genetic Advances

**DOI:** 10.3390/cancers15133373

**Published:** 2023-06-27

**Authors:** Jaffar Khan, Asad Ullah, Mya Goodbee, Kue Tylor Lee, Abdul Qahar Khan Yasinzai, James S. Lewis, Hector Mesa

**Affiliations:** 1 Department of Pathology and Laboratory Medicine, Indiana University School of Medicine, Indianapolis, IN 46202, USA; 2 Department of Pathology, Microbiology, Immunology Vanderbilt University Medical Center, Nashville, TN 37232, USA; 3 Medical College of Georgia, Augusta, GA 30912, USA; 4 Bolan Medical College, Quetta 83700, Pakistan; 5 Department of Otolaryngology-Head and Neck Surgery, Vanderbilt University Medical Center, Nashville, TN 37232, USA

**Keywords:** acinic cell carcinoma, salivary gland, SEER program, risk factors, prognostic factors, grade

## Abstract

**Simple Summary:**

Acinic cell carcinoma (AciCC) is a rare subtype of salivary gland neoplasm accounting for 6–7% of such tumors. In our study, we analyzed 2226 total cases of AciCC while conducting analysis on the demographic trends and outcomes related to different treatment strategies, clinical characteristics, and other demographics. AciCC mainly affected white females in their 50s. Grade III (poorly differentiated) and Grade IV (undifferentiated) cancers along with tumors that metastasized were associated with worse survival outcomes. This study aims to provide further data on treatment strategies, demographic features and trends, and factors associated with poorer prognosis.

**Abstract:**

Background: Acinic cell carcinoma (AciCC) comprises 6–7% of all salivary gland neoplasms and is the second most common salivary gland malignancy in children. Like many salivary gland carcinomas, it is considered low grade but occasionally it behaves aggressively. Understanding the risk factors associated with recurrence, metastasis, and death is important to determine the counseling and management of individual patients. Older population-based studies are presumed to have been confounded by the misclassification of other neoplasms as AciCC, in particular secretory carcinoma and cystadenocarcinoma. Since diagnostic tools to reliably separate these entities have been available for over a decade, reevaluation of epidemiologic data limited to the 21st century should allow a better characterization of the clinicopathological characteristics of AciCC. Methods: Our study extracted data from the Surveillance, Epidemiology, and End Results (SEER) database for the period 2000 to 2018. Cox regression model analysis was performed to identify risk factors independently affecting survival. Results: Data for 2226 patients with AciCC were extracted from the database. Most patients were females: 59%, and white: 80.5%, with a mean age at diagnosis of 51.2 (SD ± 18.7) years. Most cases (81%) were localized at presentation. Tumor size was less than 2 cm in 42%, 2–4 cm in 47%, and >4 cm in 11%. Low-grade tumors had 5-year survival > 90%, whereas high-grade tumors had survival < 50%. Of the patients with known lymph node status only 7.3% had nodal metastases. Distant metastases were documented in 1.1%, involving lungs 44%, bone 40%, liver 12%, and brain 4%. The most common treatment modality was surgery alone: 63.6% followed by surgery and adjuvant radiation: 33%. A few received chemotherapy (1.8%) or multimodality therapy (1.2%). The 5-year overall survival rate was 90.6% (95%CI 89.1–91.9), and disease-specific survival was 94.6% (95%CI 93.3–95.6). Multivariable cox regression analysis showed that undifferentiated (HR = 8.3) and poorly differentiated tumor grade (HR = 6.4), and metastasis (HR = 5.3) were the worst independent prognostic factors. Other poor risk factors included age > 50 (HR = 3.5) and tumor size > 4 cm (HR = 2.5). Conclusions: In the US, AciCC is more common in middle age white females, and most tumors are less than 4 cm and localized at diagnosis. The most relevant negative prognostic factor was high tumor grade which was associated with higher hazard ratios for death than all other variables, including regional or distant metastases at presentation.

## 1. Introduction

Acinic cell carcinoma (AciCC) accounts for 6–7% of all salivary gland malignancies, with >90% of the cases arising in the parotid gland [1]. It is the second most common salivary gland malignancy in children, but adult cases still vastly outnumber pediatric cases [2,3]. Morphologically, AciCC resembles the serous acinar cells of the salivary gland [1] and is characterized by cells with abundant cytoplasm with basophilic zymogen granules. Its morphology is highly variable though and may overlap with other salivary gland tumors in limited biopsies. However, advances in immunohistochemistry and molecular genetics that have been available for at least a decade currently allow a reliable separation of AciCC from mimickers, especially secretory carcinoma, and cystadenocarcinoma. Older population-based studies are known to have been confounded by the misclassification of other neoplasms as AciCC. Reevaluation of epidemiologic data limited to the 21st century should allow a better characterization of the clinicopathological factors affecting the prognosis of this rare malignancy.

## 2. Methods

Data from patients with a diagnosis of AciCC for the period 2000 to 2018 were obtained from the Surveillance Epidemiology and End Result (SEER) database. This database contains around 18% of the total United States population in 18 different registries: Alaska Native Tumor Registry, Arizona Indians Tumor Registry, Cherokee Nation Tumor Registry, Connecticut tumor registry, Detroit tumor registry, Georgia Center for Cancer registry, Greater Bay Area Cancer tumor Registry, Greater California registry, Hawaii Tumor Registry, Iowa Tumor Registry, Kentucky Tumor Registry, Louisiana Tumor Registry, New Jersey Tumor Registry, Seattle-Puget Sound Tumor Registry, and Utah Tumor Registry from SEER software (https://seer.cancer.gov/seerstat/, accessed on 5 March 2022). The data were exported to Statistical Product and Service Solutions (SPSS©) version 20.2 (IBM Corporation, Armonk, NY, USA). Extracted data included age, sex, and race, tumor location, size, grade, staging, treatment type, and survival time. Cases that were not microscopically confirmed were excluded. Endpoints examined in this study included overall survival and mortality at 1, 2, 3, 4, and 5 years.

Staging in the SEER database differs from the AJCC staging system. Restaging of the cases according to the AJCC staging system was not done to prevent distorting the data integrity, however, all the staging parameters used in the AJCC system such as histological grade, tumor size, and metastases were set as individual variables. IBM SPSS^®^v28.0.0.0(190) software was utilized to perform multivariate analysis on various factors affecting survival and to create Kaplan-Meier survival curves. Analysis of variance (ANOVA) was performed to identify statistically significant independent variables for the Cox regression model; for this step significance was set at *p*-value of 0.25. Multivariate Cox regression analysis was used to calculate hazard ratios for the identified independent factors affecting survival. Statistical significance was set at *p*-value of <0.05.

## 3. Results

### 3.1. Demographic Characteristics

The cohort consisted of 2226 patients with a mean age of 51 years (range 5–85 years). Seventy-eight (3.5%) were pediatric patients (≤18 years). Females comprised 59% for a F:M ratio of 1.4:1. By race 80.5% were White, 9.3% African America and 8.4% Asian or Pacific Islanders, which essentially reflects the ethnic distribution of the general US population (Table 1).

**Figure 1:**
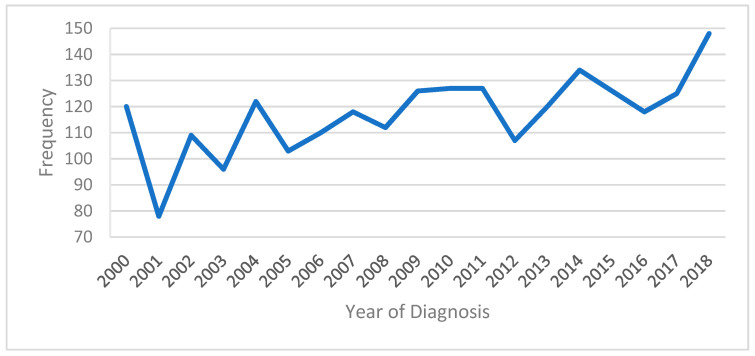
Trend analysis of patients diagnosed with AciCC from 2000 to 2018.

Between 2000 and 2018, the number of yearly diagnosed patients increased overall, except in 2001. (Figure 1).

### 3.2. Tumor Characteristics

Tumor grading had 4 categories: well-, moderately-, and poorly differentiated, and undifferentiated. Staging was classified into localized, regional, and distant. Localized was defined as confined to the organ or anatomic subsite of origin. Regional was defined as spread beyond the organ of origin either through direct extension into the surrounding tissues, lymph node metastasis, or both, and regional not otherwise specified. Distant was defined as spread to tissues/organs remote from the primary tumor. Unknown included cases with insufficient information to categorize into one of the previously defined stages (https://training.seer.cancer.gov/staging/systems/summary/regionalized.html, accessed on 15 February 2023).

Tumor grade was available in 836 patients: 49.6% were well-differentiated, 36.4% were moderately-differentiated, 8.3% were poorly-differentiated and 5.7% were undifferentiated. Tumor stage was available for 2010 (90%) patients. Localized stage was most common: 80.6%, followed by regional: 13.5%, and distant: 5.8%. Tumor size was available for 1317 (59.2%) patients: size < 2 cm: 41.6%, size 2–4 cm: 46.9% and >4 cm: 11.5%. Lymph node status was available for 1426 (64.1%) patients. Of these 7.3% had nodal metastases. Only 25 patients (1.1%) had distant metastases. The most common sites were the lung: 44%, bone 40%, liver 12% and brain 4% (Table 2).

### 3.3. Treatment Characteristics

Treatment information was available for 2101 (94%) patients: 1337 (63.6%) underwent surgery alone, 695 (33.1%) had surgery and adjuvant radiation, 38 (1.8%) received chemotherapy and 31 (1.5%) multimodality surgery, radiation, and chemotherapy (Table 3).

### 3.4. Survival by Age, Sex, and Race

**Figure 2:**
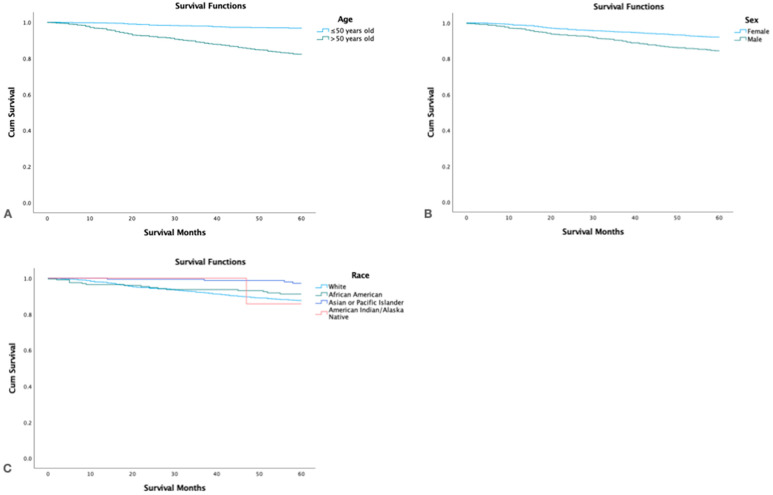
Kaplan Meier survival graphs by (**A**): Age, (**B**): Gender, (**C**): Race.

Females had a significantly higher 5-yr. survival rate than males: 96.3 vs. 91.9% (*p* < 0.05). White and Black patients had similar 5-year survival rates: 93.8 and 95.7, respectively, and American Indian, Asians, and Pacific Islanders had the highest 5-year survival rate at 99.2%, however, this difference was not significant, and the confidence intervals of all groups overlapped (Table 4, Figure 2).

### 3.5. Survival by Treatment Modality

**Figure 3:**
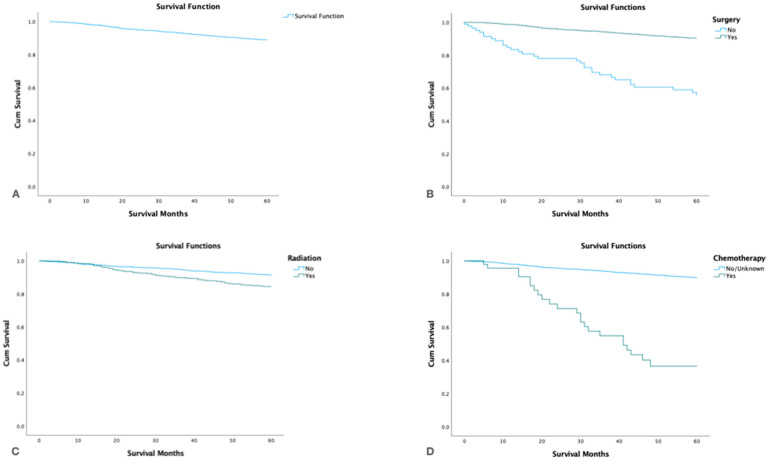
Kaplan Meier survival graphs by treatment modality: (**A**): Survival of the entire cohort. (**B**). Survival by surgery. (**C**). Survival by radiotherapy. (**D**). Survival by chemotherapy.

The overall 5-year survival rate was 90.6% (95%CI 89.1–91.9) and the cause-specific survival was 94.6% (95%CI 93.3–95.6). By treatment modality patients who underwent surgery alone had the highest 5-year survival rate 95.0% (95%CI 93.7–96.0) followed by surgery and adjuvant radiation 89.8% (95%CI 87.0–92.0). Patients who underwent only radiation or chemotherapy alone had the lowest 5-year survival (Table 5, Figure 3).

### 3.6. Survival by Tumor Grade, Stage, and Size

**Figure 4:**
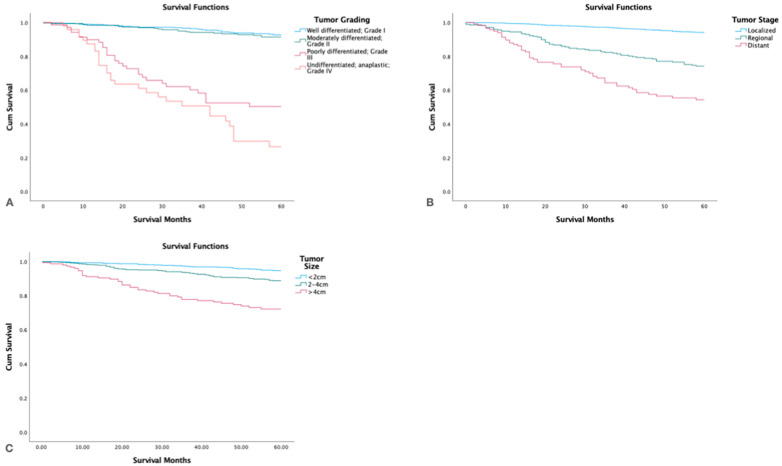
Kaplan-Meier survival graph by (**A**): Tumor Grade, (**B**): Tumor Stage, (**C**): Tumor Size.

By tumor grade, no significant differences in survival were found between patients with well-differentiated (5-yr. survival 92.7%, 95%CI 91.3–94.1) and moderately differentiated (5-yr. survival 91.4, 95%CI 89.6–93.2) tumors. However, patients with poorly differentiated and undifferentiated tumors had much worse survival: 50.3% (95% CI 43.7–56.9) and 26.5% (95% CI 19.1–33.9), respectively (*p <* 0.05). Patients with localized tumor stage had the highest 5-yr. survival (*p <* 0.05) and patients with distant spread had the lowest. Tumor size >4 cm had the lowest survival, and tumor size < 2 cm the highest (Figure 4).

**Figure 5:**
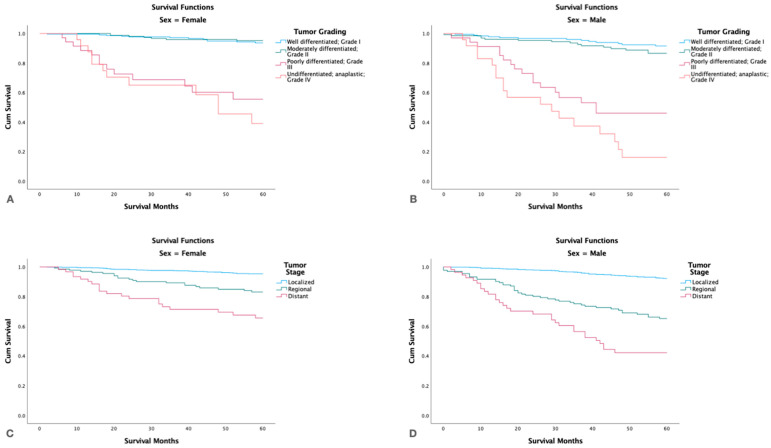
Kaplan-Meier survival graphs by gender, tumor grade and stage. (**A**,**B**): Survival in females and males by tumor grade. (**C**,**D**): Survival in females and males by tumor stage.

Stratifying by grade and gender showed that males with high tumor grade had lower survival rates than females (*p* < 0.001). Males also had lower survival rates than females in all stages (localized, regional, distant) (*p <* 0.001) (Figure 5).

### 3.7. Survival with Distant Metastasis

**Figure 6:**
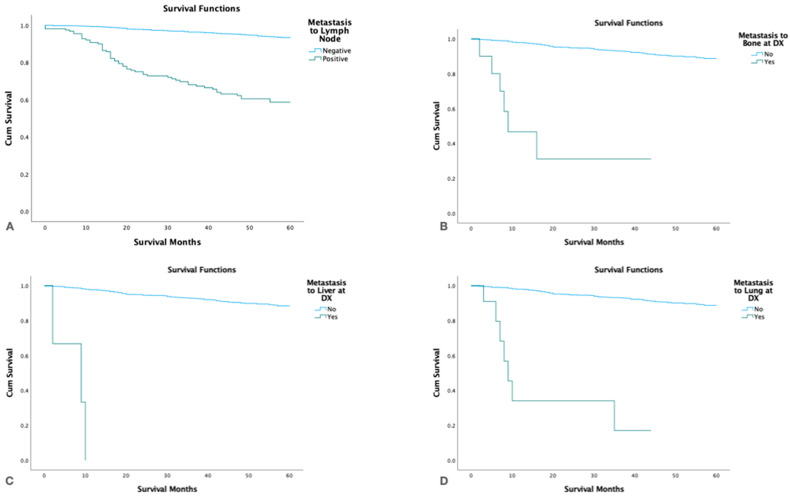
Kaplan Meier survival graphs by metastatic site at the time of diagnosis. (**A**). Lymph nodes, (**B**). Bone, (**C**). Liver, (**D**). Lungs.

Patients with distant metastasis had significantly lower 5-yr. survival (*p* < 0.05) than patients without distant spread and patients with positive lymph nodes also had significantly lower 5-yr. survival than node-negative patients (*p* < 0.001). Patients with liver metastasis had shorter survival than patients with lung and bone metastases (Figure 6).

**Figure 7:**
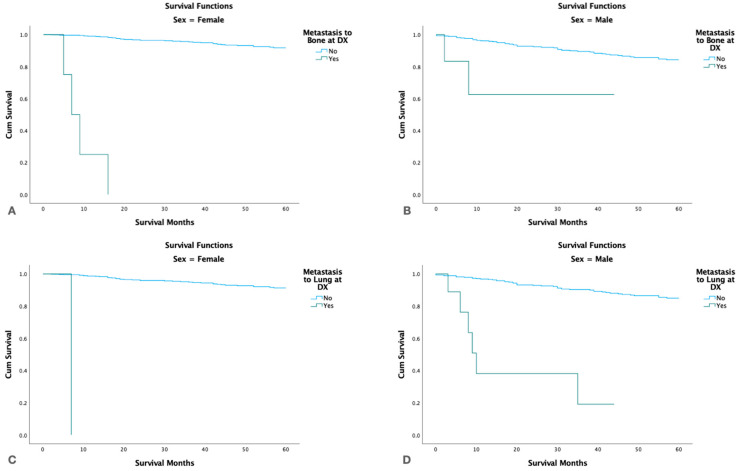
Kaplan-Meier survival graphs for patients with metastasis at time of diagnosis stratified by gender. (**A**,**B**): Survival in females and males with metastasis to bone. (**C**,**D**): Survival in females and males with metastasis to lung.

Stratified by gender, males with bone or liver metastasis had a significantly higher survival than females (*p* < 0.001) (Figure 7).

### 3.8. Multivariate Analysis

ANOVA was conducted to identify independent variables to be used for the Cox regression model with significance set at *p* < 0.25. The results of the Cox regression analysis revealed that poorly differentiated (HR 6.4, 95%CI 3.1–13.1) and undifferentiated tumor grade (HR 8.3, 95%CI 4.0–17.2) were the factors demonstrating the strongest association with decreased survival. Other independent factors included distant spread (HR 5.3, 95%CI 2.8–10.2), age > 50 years (HR 3.5, 95%CI 1.8–6.7) and tumor size > 4 cm (HR 2.5, 95%CI 1.3–4.9) (Table 6).

## 4. Discussion

Our study using the SEER database for the period 2000–2018 shows that in patients with AciCC, the risk factors associated with the worst outcomes in order of importance were tumor grade, distant metastasis, age > 50 years, and tumor size > 4 cm. Kaya et al. performed a SEER database study for the period 1975–2016 and also found that younger age, female sex, earlier stage, low grade, and surgical management predicted a better outcome in patients with AciCC [4]. A study by Quinn et al. in 2018 using the National Cancer Database (NCDB) reported that primary lymph node involvement, especially if >1 node and tumor size had a stronger negative impact on survival than other factors, however, tumor grade was not analyzed [5]. In our study, nodal metastases and size were associated with statistically significant lower 5-yr survival, however, their impact was smaller than tumor grade, distant metastases, and age > 50 years. A study by Scherl et al. from 2018 also using the NCDB reported that in order of importance, age > 70 years, high-grade histology, pathologic stage N2 (multiple ipsilateral nodes or extranodal extension), T4 (advanced disease), and tumor size > 6 cm were associated with the worst outcomes [6].

The recent findings, combined with our own study, provide clear evidence that high-grade transformation is a significant predictor of mortality in AciCC. While large database studies lack detailed information on how grading is applied to individual patients, retrospective single institutional cohorts with well-defined criteria for high-grade AciCC demonstrate its importance. Chintakuntlawar et al. reported that among patients with AciCC and high-grade transformation, those with a mitotic activity > 4 per 10 high-power fields and/or necrosis experienced markedly worse survival. Similarly, Xu et al., utilizing the same mitotic threshold to define high-grade transformation, observed significantly poorer overall and disease-specific survival in these patients. All of these studies consistently lead to the same conclusion: high histologic grade is the most powerful predictor of outcome, surpassing other clinical or pathologic factors, including the presence of distant metastasis at presentation [7,8].

The current WHO classification of salivary gland tumors and the 8th edition of the AJCC Cancer Staging System do not have a formal grading system for AciCC but include the presence or absence of “high grade transformation” as a required parameter to be reported [9,10]. According to the WHO (World Health Organization), high-grade transformation (or high grade) in AciCC is described as the presence of morphologically distinct areas within the tumor, exhibiting marked cytologic atypia, increased mitotic activity, atypical mitoses, tumor necrosis, and clinical features such as rapid progression and facial nerve involvement [11,12]. However, this definition lacks the clarity provided by the simpler definition used by Xu et al. and Chintakuntlawar et al., which defines high grade based on a mitotic activity of ≥ 4 per 10 high-power fields and/or the presence of necrosis. By adopting this simpler definition, there is no need for a separate categorization of “dedifferentiated,” “undifferentiated,” or “high-grade transformed” AciCC, streamlining the classification approach.

In our study, only a small number of cases had distant metastasis (1.1%). The most common sites were lung, bone, liver, and brain. Patients with liver metastasis had shorter survival than patients with lung and bone metastases. These results may reflect the ability to provide adjuvant radiation or perform excision at the different sites. Stratified by gender, males with bone or liver metastasis were found to have a significantly higher survival in comparison to females. This could reflect gender-associated biologic variations in the ability to tolerate adjuvant therapy or less likely, gender-associated differences in the ability/eligibility or willingness to undergo adjuvant therapies at advanced stages.

In our study, it was found that 7% of the patients with AciCC had nodal metastasis at the time of diagnosis. This finding is consistent with the nodal metastasis rates reported in other studies, which range from 8% to 9% [2]. However, when comparing these results with studies using the National Cancer Database (NCDB), a higher incidence of lymph node metastases, ~22%, has been reported [6,13,14]. This discrepancy in nodal metastasis rates between studies using the SEER and the NCDB databases highlights the importance of considering variations in patient populations and data sources when interpreting and comparing findings across different studies. Some studies report that nodal metastasis affects mostly patients who have persistent or recurrent disease [1,2]. Fang et al. reported that positive intra-parotid lymph nodes in patients with negative neck nodes increased the risk for local recurrence and distant metastases; this interesting finding, which includes real angiolymphatic spread and extension by contiguity, has yet to be confirmed by others [15]. Our study findings demonstrate that the majority of patients with AciCC present with localized tumors, supporting the notion that surgery alone yields superior outcomes, particularly for early-stage tumors. These results also reaffirm the indolent nature and slow progression of AciCC, as well as its low incidence of regional and distant metastasis. Consequently, they provide a rationale for the current recommendation of complete resection of primary tumors and surgical intervention for recurrent or oligometastatic disease when feasible [1,16]. When analyzing the five-year survival rates based on tumor grade in our study, grades I and II exhibited survival rates exceeding 90%, whereas grades III and IV had significantly lower rates (50% and 26%, respectively). These findings underscore the prognostic significance of tumor grade, with higher-grade tumors associated with poorer survival outcomes.

For unresectable, incompletely resected, or completely resected tumors with high-risk features adjuvant radiotherapy is usually administered. High-risk features include high-grade transformation, perineural invasion, angiolymphatic invasion and extra-nodal extension [17,18].

Chemotherapy is uncommon and reserved for patients with unresectable or disseminated disease, and usually administered in the context of clinical trials. Platinum agents and cetuximab are commonly used [19].

The retrospective study conducted by Yibulayin et al. provided evidence supporting the association between high tumor grade and a poorer prognosis, while highlighting the improved disease-free and overall survival outcomes associated with surgical intervention [18,20]. Similarly, Biron et al. observed similar results, where high tumor grade emerged as a stronger predictor of survival compared to TNM staging [21]. These findings align with the results of our study, further validating the significance of tumor grade as a prognostic factor in AciCC.

### 4.1. Recent Molecular Genetic Advances

A recurrent translocation t (4:9) (q13; q31) was demonstrated in >90% of AciCC by Haller et al. [22]. The translocation involves the enhancer *secretory Ca-binding phosphoprotein (SCPP)* in chromosome 4q13 to the upstream region of the transcription factor *Nuclear Receptor Subfamily 4 Group A Member 3 (NR4A3)* in 9q31, resulting in constitutive upregulation of *NR4A3*, but not a chimeric fusion protein. *NR4A3* abnormalities have also been described in extra-skeletal myxoid chondrosarcoma (*EWSR1/NR4A3* fusion) and targeted therapies for this gene are not currently available [23]. Haller et al. also found that overexpression of NR4A3 protein in AciCC can be demonstrated by immunohistochemistry (IHC) with a commercially available antibody with sensitivity and specificity approaching 100% [22]. A small subset of AciCC cases (4–8%) has an alternative fusion involving the *Histatin 3 and Myb/SANT-like DNA-binding domain containing 3* genes *(HTN3-MSANTD3).* The fusion also causes overexpression of NR4A3 by IHC indicating an alternative pathway that results in upregulation of this gene, further supporting its role as an oncogenic driver in AciCC.

In a recent study using comprehensive genomic profiling of metastatic and relapsed salivary gland tumors, Ross et al. found frequent genomic alterations in *CDKN2A* (76%), *CDKN2B* (45%), which are not currently targetable, and less frequent alterations in *P53* (9%), *PTEN* (9%), *FBXW7* (8%), *ATM* (7%), *BRAF* (5%), and *NF1* (5%) [24]. Of these, *PTEN* and *NF1* alterations should be susceptible to mTOR pathway inhibitors, and among the *BRAF* mutated cases, the specific *BRAF V600E* mutation should respond to BRAF inhibitors. Overall, AciCC showed a low mutational burden predicting low response to immunotherapy and no amplification of *HER2* preventing the use of anti-HER2 therapy [25]. As mentioned before, the translocation of the active enhancer regions from the *SCPP* gene cluster to the upstream region of *NR4A3* leads to upregulation of the latter through enhancer hijacking, leading to oncogenic effects and tumor proliferation [26,27,28].

### 4.2. Limitations

The limitations of this study are common to large database studies and are related to missing or incomplete information, misclassification of variables, and errors in the information entered in the database. Low-quality or incomplete data is most likely to affect low-incidence variables. It also suffers from lack of consistent definitions, particularly as it relates to tumor grade, and the change over time in tumor classification. SEER does not provide information about the specific type of surgery and indications regarding the surgery. Secretory carcinoma, which was probably misclassified as AciCC before it was clearly recognized as a distinct entity in 2010, must certainly be “hiding” in our cohort of AciCC from the early 2000s [29].

## 5. Conclusions

AciCC is a generally indolent salivary gland carcinoma affecting mostly middle-aged adults, with a slight female preponderance. Most cases are localized at diagnosis and are cured by surgical excision. The most important negative prognostic risk factors encountered are high histologic grade (HR: 6.4–8.3), distant spread (HR: 5.3), age > 50 years (HR: 3.5) and tumor size > 4 cm (HR 2.5). Complete resection of resectable tumors at the earliest possible time, adjuvant radiation and close surveillance for patients with high-risk features, and resection of recurrences and oligometastatic disease represent the current standard of care for patients with AciCC. For advanced-stage tumors, comprehensive genomic analysis for the identification of potential therapeutic targets and chemotherapy in the context of a clinical trial are the only available options at this time.

## Figures and Tables

**Table 1:** Demographics and Clinical Profile of Patients with AciCC from SEER database 2000–2018.

Variable	N (%)
**Total Subjects**	2226 (100.0)
**Age in years** (Mean ± SD)	51.2 ± 18.7
**Sex** **n (%)**	
Male	912 (41.0)
Female	1314 (59.0)
**Race** **n (%)**	
White	1791 (81.6)
African American	206 (9.4)
Asian or Pacific Islander	188 (8.6)
American Indian/Alaskan Native	10 (0.5)

**Table 2:** Tumor Characteristics and Stage of AciCC Patients from the SEER database 2000–2018.

Tumor Grade (N = 836) n (%)	
Well Differentiated	415 (49.6)
Moderately Differentiated	304 (36.4)
Poorly Differentiated	69 (8.3)
Undifferentiated	48 (5.7)
**Tumor Stage (N = 2010)** **n (%)**	
Localized	1621 (80.6)
Regional	272 (13.5)
Distant	117 (5.8)
**Tumor Size (N = 1317)**	**n (%)**
<2 cm	547 (41.5)
2–4 cm	619 (47.0)
>4 cm	151 (11.5)
**Lymph Node Status (N = 1426)** **n (%)**	
Positive N (%)	162 (11.4)
Negative N (%)	1264 (88.6)
**Distant Metastasis (N = 25)** **n (%)**	
Lung N (%)	11 (44.0)
Bone N (%)	10 (40.0)
Liver N (%)	3 (12.0)
Brain N (%)	1 (4.0)

**Table 3:** Treatment modality for patients with AciCC from the SEER database 2000–2018.

Treatment (N = 2101)	n (%)
Surgery Only	1337 (63.6)
Radiation(RT) After Surgery	695 (33.1)
Chemotherapy (QT)	38 (1.8)
Combined Surgery, RT, and QT	31 (1.5)

**Table 4:** Overall survival data by gender and race.

Survival	Male % (95%CI)	Female % (95%CI)	White % (95%CI)	Black % (95%CI)	American Indian/Asians/Pacific Islander % (95%CI)
1 year	98.8 (97.6–99.4)	99.3 (98.6–99.7)	99.2 (98.5–99.5)	97.7 (94.0–99.1)	100
2 years	97.0 (95.5–98.0)	97.9 (96.8–98.6)	97.3 (96.3–98.0)	97.1 (93.1–98.8)	100
3 years	94.8 (92.9–96.3)	97.3 (96.0–98.1)	95.8 (94.6–96.8)	96.4 (92.3–98.4)	100
4 years	93.2 (90.9–94.9)	96.8 (95.5–97.7)	94.7 (93.3–95.8)	96.4 (92.3–98.4)	99.2 (94.7–99.9)
5 years	91.9 (89.5–93.8)	96.3 (94.9–97.3)	93.8 (92.3–95.0)	95.7 (91.2–98.0)	99.2 (94.7–99.9)

**Table 5:** Survival data of 2226 Patients with AciCC from the SEER Database 2000–2018, stratified by therapy.

Survival	Overall 5-Year. % (95%CI)	Cause Specific 5-Year. % (95%CI)	Surgery (S) % (95%CI)	S and Radiation (RT) % (95%CI)	Chemotherapy (QT) % (95%CI)	Combined S, RT, and QT % (95%CI)
1 year	98.5 (97.8–98.9)	99.1 (98.6–99.5)	99.4 (98.9–99.7)	99.5 (98.6–99.9)	100.0	100.0
2 years	96.0 (95.0–96.8)	97.6 (96.7–98.2)	97.9 (97.1–98.5)	96.1 (94.3–97.4)	81.7 (61.4–92.0)	77.4 (54.0–89.9)
3 years	94.2 (93.0–95.2)	96.3 (95.3–97.1)	96.8 (95.8–97.6)	93.7 (91.4–95.4)	63.1 (42.3–78.2)	63.8 (40.4–80.0)
4 years	92.4 (91.1–93.6)	95.4 (94.2–96.3)	95.8 (94.7–96.7)	91.8 (89.2–93.8)	46.3 (26.5–64.0)	44.1 (23.0–63.5)
5 years	90.6 (89.1–91.9)	94.6 (93.3–95.6)	95.0 (93.7–96.0)	89.8 (87.0–92.0)	41.7 (22.5–59.9)	38.6 (18.4–58.5)

**Table 6:** Multivariate Overall Survival Analysis.

Variables	Univariate Analysis	Multivariate Analysis	
	ANOVA F Value (*p*-Value)	Hazard Ratio (*p*-Value)	95% Confidence Interval
Undifferentiated	27.3 (<0.001)	8.3 (<0.001)	4.0–17.2
Poorly Differentiated	27.3 (<0.001)	6.4 (<0.001)	3.1–13.1
Distant Metastasis	30.7 (<0.001)	5.3 (<0.001)	2.8–10.2
Age > 50	26.4 (<0.001)	3.5 (<0.001)	1.8–6.7
Tumor Size > 4 cm	28.1 (<0.001)	2.5 (0.006)	1.3–4.9

## Data Availability

All data are publicly available.

## References

[1] Cavaliere M., De Luca P., Scarpa A., Savignano L., Cassandro C., Iemma M. (2020). Acinic cell carcinoma of the parotid gland: From pathogenesis to management: A literature review. Eur. Arch. Oto-Rhino-Laryngol..

[2] Neskey David M., Klein Jonah D., Hicks S., Garden Adam S., Bell Diana M., El-Naggar Adel K., Kies Merrill S., Weber Randal S., Kupferman Michael E. (2013). Prognostic Factors Associated with Decreased Survival in Patients with Acinic Cell Carcinoma. JAMA Otolaryngol.–Head Neck Surg..

[3] Khosravi M.H., Bagherihagh A., Saeedi M., Dabirmoghaddam P., Kouhi A., Amirzade-Iranaq M.H. (2017). Salivary Gland Cancers: A Survey through History, Classifications and Managements. Diagnosis and Management of Head and Neck Cancer.

[4] Kaya E.A., Taylor Z.C., Mitchell B.J., Guss Z.D., Bunn J.D., Fairbanks R.K., Lamoreaux W.T., Wagner A.E., Peressini B.J., Lee C.M. (2020). Clinicopathologic Features and Survival Trends for Acinic Cell Carcinoma of the Major Salivary Glands: A Surveillance, Epidemiology, and End Results Population Analysis. World J. Oncol..

[5] Quinn C., Robbins J.R., Shukla M.E., Firat S., Massey B., Schultz C.J., Wong S., Campbell B., Stadler M. (2018). Acinic Cell Carcinoma of the Major Salivary Glands: Analysis of Prognostic Factors in 2950 patients. Int. J. Radiat. Oncol. Biol. Phys..

[6] Scherl C., Kato M.G., Erkul E., Graboyes E., Nguyen S.A., Chi A.C., Morgan P., Day T.A. (2018). Outcomes and prognostic factors for parotid acinic cell Carcinoma: A National Cancer Database study of 2362 cases. Oral Oncol..

[7] Chintakuntlawar A.V., Shon W., Erickson-Johnson M., Bilodeau E., Jenkins S.M., Davidson J.A., Keeney M.G., Rivera M., Price D.L., Moore E.J. (2016). High-grade transformation of acinic cell carcinoma: An inadequately treated entity?. Oral Surg. Oral Med. Oral Pathol. Oral Radiol..

[8] Xu B.M., Saliba M., Ho A., Viswanathan K., Alzumaili B., Dogan S., Ghossein R., Katabi N. (2022). Head and Neck Acinic Cell Carcinoma: A New Grading System Proposal and Diagnostic Utility of NR4A3 Immunohistochemistry. Am. J. Surg. Pathol..

[9] WHO Classification of Tumours Editorial Board (2022). Head and Neck Tumours. Lyon (France): International Agency for Research on Cancer. WHO Classification of Tumours Series.

[10] Amin M.B., Edge S.B., Greene F.L., Byrd D.R., Brookland R.K., Washington M.K., Gershenwald J.E., Compton C.C., Hess K.R., Sullivan D.C. (2017). AJCC Cancer Staging Manual.

[11] Skalova A., Sima R., Vanecek T., Muller S., Korabecna M., Nemcova J., Elmberger G., Leivo I., Passador-Santos F., Walter J. (2009). Acinic cell carcinoma with high-grade transformation: A report of 9 cases with immunohistochemical study and analysis of TP53 and HER-2/neu genes. Am. J. Surg. Pathol..

[12] Thompson L.D., Aslam M.N., Stall J.N., Udager A., Chiosea S., McHugh J. (2016). Clinicopathologic and immunophenotypic characterization of 25 cases of acinic cell carcinoma with high-grade transformation. Head Neck Pathol..

[13] Hoffman H.T., Karnell L.H., Robinson R.A., Pinkston J.A., Menck H.R. (1999). National Cancer Data Base report on cancer of the head and neck: Acinic cell carcinoma. Head Neck.

[14] Xiao C.C., Zhan K.Y., White-Gilbertson S.J., Day T.A. (2016). Predictors of Nodal Metastasis in Parotid Malignancies. Otolaryngol. Neck Surg..

[15] Fang Q., Wu J., Du W., Zhang X. (2019). Predictors of distant metastasis in parotid acinic cell carcinoma. BMC Cancer.

[16] Pang Y., Sun L., Liu H., Ma J. (2021). Differential diagnosis and treatment of salivary secretory carcinoma and acinic cell carcinoma. Oral Oncol..

[17] Park Y.M., Yoon S.O., Kim J.H., Kang M.S., Kim D.H., Koh Y.W., Kim S.-H., Lim J.-Y., Choi E.C. (2021). Comprehensive Analysis of Clinicopathologic Factors Predictive of an Unfavorable Prognosis in Patients with Acinic Cell Carcinoma of the Parotid Gland. Clin. Exp. Otorhinolaryngol..

[18] Gomez Daniel R., Katabi N., Zhung J., Wolden Suzanne L., Zelefsky Michael J., Kraus Dennis H., Shah Jatin P., Wong Richard J., Ghossein Ronald A., Lee Nancy Y. (2009). Clinical and pathologic prognostic fea-tures in acinic cell carcinoma of the parotid gland. Cancer.

[19] Mueller S.K., Haderlein M., Lettmaier S., Agaimy A., Haller F., Hecht M., Fietkau R., Iro H., Mantsopoulos K. (2022). Targeted Therapy, Chemotherapy, Immunotherapy and Novel Treatment Options for Different Subtypes of Salivary Gland Cancer. J. Clin. Med..

[20] Yibulayin F., Feng L., Wang M., Lu M.M., Luo Y., Liu H., Yang Z.C., Wushou A. (2020). Head & neck acinar cell carcinoma: A population-based study using the seer registry. BMC Cancer.

[21] Biron V.L., Lentsch E.J., Gerry D.R., Bewley A.F. (2015). Factors influencing survival in acinic cell carcinoma: A retrospective survival analysis of 2061 patients. Head Neck.

[22] Haller F., Skálová A., Ihrler S., Bruno M., Bieg M., Moskalev E.A., Erber R., Blank S., Winkelmann C., Hebele S. (2019). Nuclear NR4A3 Immunostaining Is a Specific and Sensitive Novel Marker for Acinic Cell Carcinoma of the Salivary Glands. Am. J. Surg. Pathol..

[23] Filion C., Motoi T., Olshen A.B., Laé M., Emnett R.J., Gutmann D.H., Perry A., Ladanyi M., Labelle Y. (2009). The EWSR1/NR4A3 fusion protein of extraskeletal myxoid chondrosarcoma activates the PPARG nuclear receptor gene. J. Pathol..

[24] Ross J.S., Gay L.M., Wang K., Vergilio J.A., Suh J., Ramkissoon S., Somerset H., Johnson J.M., Russell J., Ali S. (2017). Comprehensive genomic profiles of metastatic and relapsed salivary gland carcinomas are as-sociated with tumor type and reveal new routes to targeted therapies. Ann. Oncol..

[25] Porcheri C., Meisel C.T., Mitsiadis T.A. (2020). Molecular and Cellular Modelling of Salivary Gland Tumors Open New Landscapes in Diagnosis and Treatment. Cancers.

[26] Haller F., Bieg M., Will R., Körner C., Weichenhan D., Bott A., Ishaque N., Lutsik P., Moskalev E.A., Mueller S.K. (2019). Enhancer hijacking activates oncogenic transcription factor NR4A3 in acinic cell carcinomas of the salivary glands. Nat. Commun..

[27] Herz H.M. (2016). Enhancer deregulation in cancer and other diseases. Bioessays.

[28] Gröschel S., Sanders M.A., Hoogenboezem R., de Wit E., Bouwman B.A.M., Erpelinck C., van der Velden V.H.J., Haver-mans M., Avellino R., van Lom K. (2014). A single oncogenic enhancer rearrangement causes concomi-tant EVI1 and GATA2 deregulation in leukemia. Cell.

[29] Skalova A. (2013). Mammary Analogue Secretory Carcinoma of Salivary Gland Origin: An Update and Expanded Morphologic and Immunohistochemical Spectrum of Recently Described Entity. Head Neck Pathol..

